# A diphenyldiselenide derivative induces autophagy *via* JNK in HTB‐54 lung cancer cells

**DOI:** 10.1111/jcmm.13318

**Published:** 2017-09-18

**Authors:** Marta Díaz, Roncesvalles González, Daniel Plano, Juan Antonio Palop, Carmen Sanmartín, Ignacio Encío

**Affiliations:** ^1^ Department of Organic and Pharmaceutical Chemistry Faculty of Pharmacy and Nutrition University of Navarra Pamplona Spain; ^2^ Instituto de Investigaciones Sanitarias de Navarra (IDISNA) Pamplona Spain; ^3^ Departamento de Ciencias de la Salud Universidad Pública de Navarra Pamplona Spain

**Keywords:** p53, p38 MAPK, JNK, diphenyldiselenide, autophagy

## Abstract

Symmetric aromatic diselenides are potential anticancer agents with strong cytotoxic activity. In this study, the *in vitro* anticancer activities of a novel series of diarylseleno derivatives from the diphenyldiselenide (DPDS) scaffold were evaluated. Most of the compounds exhibited high efficacy for inducing cytotoxicity against different human cancer cell lines. DPDS **2**, the compound with the lowest mean GI_50_ value, induced both caspase‐dependent apoptosis and arrest at the G_0_/G_1_ phase in acute lymphoblastic leucemia CCRF‐CEM cells. Consistent with this, PARP cleavage; enhanced caspase‐2, ‐3, ‐8 and ‐9 activity; reduced CDK4 expression and increased levels of p53 were detected in these cells upon DPDS **2** treatment. Mutated p53 expressed in CCRF‐CEM cells retains its transactivating activity. Therefore, increased levels of p21^CIP1^ and BAX proteins were also detected. On the other hand, DPDS **6**, the compound with the highest selectivity index for cancer cells, resulted in G_2_/M cell cycle arrest and caspase‐independent cell death in p53 deficient HTB‐54 lung cancer cells. Autophagy inhibitors 3‐methyladenine, wortmannin and chloroquine inhibited DPDS **6**‐induced cell death. Consistent with autophagy, increased LC3‐II and decreased SQSTM1/p62 levels were detected in HTB‐54 cells in response to DPDS **6**. Induction of JNK phosphorylation and a reduction in phospho‐p38 MAPK were also detected. Moreover, the JNK inhibitor SP600125‐protected HTB‐54 cells from DPDS **6**‐induced cell death indicating that JNK activation is involved in DPDS **6**‐induced autophagy. These results highlight the anticancer effects of these derivatives and warrant future studies examining their clinical potential.

## Introduction

Epidemiological and experimental data indicate that selenium (Se) protects against several types of cancer, including leucemia, breast, colon, lung and prostate cancer 1, 2, 3. Se provides antioxidant protection and disarms several hallmarks of cancer, such as deregulated cancer metabolism, cancer‐associated immunosuppression, regulation of cell proliferation and tumour cell invasion and angiogenesis 4. In this context, a major challenge for chemical engineering has been the development of novel Se‐containing drugs for the treatment of cancer. Thus, sodium selenite 5, 6, 7, 8, methylseleninic acid 9, selenomethionine 10, methylselenocarbamates 11 and bisacylimidoselenocarbamates 12 have been evaluated, among others, as potential anticancer agents. A number of mechanisms, including inhibition of tumour angiogenesis, cell cycle arrest 5, 10, apoptosis induction 5, 13, modulation of autophagy 14 and mitotic catastrophe 12, account for the anticancer effects of these compounds.

Over the last years, crosstalk between distinct modes of cell death, including autophagy and apoptosis, has been reported, and p53 status has emerged as important for both of these modes of cell death. Thus, whereas basal p53 activity suppresses autophagy, p53 activation by some stimuli induces both autophagy and apoptosis 15, 16. JNK and MAPK14 (p38α) signallings also regulate autophagy 17. Depending on the cell type and the nature of the stimulus, JNK and p38α can mediate cell death or cell survival 18, 19. For instance, piperlongumine‐induced autophagy in U2OS osteosarcoma cells is dependent on the activation of the p38 pathway 20, whereas inhibition of p38 phosphorylation is required for the induction of apoptosis and autophagy by plumbaginin in PC‐3 prostate cancer cells 21. On the other hand, JNK1 acts as a positive regulator of autophagy in non‐neuronal cells, whereas inhibition of Bnip3 transcription by neuronal JNK suppress autophagy 22. Interestingly, when testing sodium selenite in leucemia 5, 6, 7 and colorectal cancer 23 cells, both p53 status of the cell and p38α phosphorylation have been implicated in the switch from autophagy to apoptosis. In fact, p38α phosphorylation promoted apoptosis and compromised autophagy in NB4 leucemia cells 7. On the other hand, selenite‐mediated p38α phosphorylation induced a marked autophagic response in p53‐deficient Jurkat cells 6. Autophagy also occurred in both p53 expressing and p53‐KO HCT‐116 colorectal cancer cells exposed to selenite. However, selenite‐induced autophagy levels were higher in the p53‐KO cells 23.

Lung cancer is the leading cause of cancer death worldwide. Approximately 80% of lung cancer cases are classified histologically as non‐small cell lung cancer (NSCLC) 24. The p53 gene is inactivated by mutation in greater than 50% of NSCLC patients, and p53 mutations are a statistically significant predictor of poor outcome in patients with stage I NSCLC 24. Interestingly, both autophagy and apoptosis play a role in NSCLC progression, and p53‐KO cells displayed enhanced autophagy compared with their wild‐type counterparts 25. Diphenyldiselenide (DPDS) is an active organoselenium compound with antioxidant, neuroprotective and anti‐inflammatory properties 26. DPDS exhibits cytotoxic activity against a number of tumour cell lines 27, 28. We aimed to analyse the anticancer potential of novel symmetrical diarylseleno derivatives from the DPDS scaffold. Here, we demonstrate that DPDS **6**, one such derivative, induced cell cycle arrest and autophagy in p53‐deficient NSCLC cells by modulating JNK and p38 MAPK activities.

## Materials and methods

### Materials

The synthesis of the compounds described in this work was performed at the Department of Organic and Pharmaceutical Chemistry of the University of Navarra. The synthesis was performed *via* a reaction between 4,4′‐diaminodiphenyldiselenide and the appropriate isocyanate (compounds DPDS **1**–**4**), isothiocyanate (compounds DPDS **5**–**9**) or isoselenocyanate (compounds **10**–**13**) in a 1:2 molar ratio, in dry dioxane. The final compounds were obtained at yields ranging from 2% to 75%. Isocyanates and isothiocyanates were commercially available, but the corresponding isoselenocyanates were prepared in two steps by formylation of amines followed by the treatment with phosgene and Se powder in the presence of triethylamine under reflux. Each product was identified by infrared, ^1^H‐NMR and ^13^C‐NMR spectroscopy, elemental analysis and mass spectrometry.

### Cell culture

Human cell lines were obtained from the American Type Culture Collection (ATCC; Manassas, VA, USA). CCRF‐CEM (T‐ALL), K‐562 (chronic myeloid leucemia), PC‐3 (prostate carcinoma), HT‐29 (colon carcinoma), HTB‐54 (lung carcinoma), MOLT‐4 (T‐ALL) and A549 (lung carcinoma) cells were grown in RPMI 1640 (Invitrogen, Carlsbad, CA, USA) supplemented with 10% foetal bovine serum (FBS). MCF‐7 (breast adenocarcinoma) cells were grown in EMEM (ATCC) supplemented with 10% FBS. In addition, 184B5 (non‐malignant, mammary‐gland derived) cells were grown in Hams F12/DMEM (50:50) supplemented as previously described 12. BEAS‐2B (non‐malignant, derived from bronchial epithelium) were grown in RPMI 1640 supplemented with 5% FBS, 1× insulin‐transferrin‐sodium selenite (ITS), 500 ng/ml hydrocortisone, 2 mM sodium pyruvate, 2 mM glutamine, 20 mg/ml penicillin/gentamicin, 20 ng/ml epidermal growth factor (EGF) and 0.3 nM retinoic acid. Media were renewed every 2 days, and cells were subcultured at a ratio of 1:3.

### Cytotoxic and antiproliferative activities

Cytotoxicity was determined using the MTT (3‐(4,5‐dimethylthiazol‐2‐yl)‐2,5‐diphenyl‐tetrazolium bromide) method at five different doses ranging from 0.01 to 100 μM, as previously described 12. Briefly, depending on the cell line, 8,000 to 40,000 cells/well were plated in 96‐well plates. Plates were incubated overnight at 37°C in a humidified atmosphere containing 5% CO_2_. Then, the media were replaced by media containing DPDSs at the appropriate concentration. DPDSs were dissolved in DMSO at a concentration of 0.1 M, and 10‐fold serial dilutions were made from this stock using complete culture medium. To measure the cell population at the time of drug addition, 20 μl of a 5 mg/ml solution of MTT in PBS was added to each well of one plate with no DPDS added. After mixing, cells were incubated for 4 additional hours to allow the MTT conversion into formazan. After incubation, media were removed. Formazan was dissolved in 200 μl DMSO, and optical density (time zero; A0) was read at 550 nm. Regarding plates containing drugs, following drug addition, the plates were incubated for an additional 72 hrs to allow the drug to take effect. After incubation, 20 μl of the MTT solution was added to each well, and plates were processed as described above. Optical densities were thus obtained for control cells (no drug added; Ac) and cells grown in the presence of DPDS at the five concentration levels (test growth; Ai). Percentage growth inhibition was then calculated as [(Ai − A0)/Ac − A0)] × 100 when Ai ≥ A0 and [(Ai − A0)/A0)] × 100 when Ai < A0. The results were obtained from at least three independent experiments performed in quadruplicate and expressed as GI_50_, which is the concentration that results in 50% reduction in the growth of treated cells with respect to untreated controls; and LC_50_, the concentration resulting in a 50% reduction of the initial number of cells.

### Cell cycle analysis and quantification of cell death

Cell cycle analysis and quantification of cell death were conducted by flow cytometry. For HTB‐54 and A549 cells, the cell death status and cell cycle analysis were determined using the Apo‐Direct kit (BD Pharmingen, BD Biosciences, Madrid, Spain) based on the TUNEL technique under the conditions described by the manufacturer. The apoptotic status of leucemia CCRF‐CEM and MOLT‐4 cells was evaluated using the Annexin V‐FITC Kit (BD Pharmingen). For apoptosis and autophagy inhibition experiments, cells were pre‐incubated for 1 hr in the absence or presence of 50 μM z‐VAD‐fmk, 10 μM wortmannin, 5 mM 3‐methyladenine (3‐MA) or 10 μM chloroquine (CQ) (Sigma‐Aldrich, St. Louis, MO, USA). To assess the role of JNK, cells were pre‐treated for 1 hr with 10 μM SP600125 (Invivogen, San Diego, CA, USA). For CCRF‐CEM and MOLT‐4 cell cycle analysis, cells were fixed in 70% ethanol, and the DNA content of fixed cells was measured by staining with propidium iodide.

### Caspase activity determination

Caspase activities were assayed using the Apo Alert Caspase Assay Plates (Clontech Laboratories, Mountain View, CA, USA) under the conditions described by the manufacturer.

### Protein analysis

Proteins were detected by Western blot. Anti‐Actin (H‐300), anti‐Cdk‐4 (C‐22), anti‐p53 (C‐19) and anti‐p21 (N‐20) antibodies were obtained from Santa Cruz Biotechnology (Santa Cruz, CA, USA). Specific antibodies for PARP, BAX, p14 ARF (4C6/4), SQSTM1/p62, LC3, p38 MAPK, Phospho‐p38 MAPK (Thr180/Tyr182), SAPK/JNK and Phospho‐SAPK (Thr183/Tyr185) (G9) detection were obtained from Cell Signaling Technology (Danvers, MA, USA). Anti‐rabbit IgG, anti‐goat IgG or anti‐mouse IgG conjugated with peroxidase were used as secondary antibodies.

### Statistical analysis

Statistical analysis was performed using SPSS 15.0 software. The two‐tailed Student's non‐parametric *t*‐test (Mann–Whitney *U*‐test) was used to compare non‐normally distributed data from two groups. Differences were considered significant at *P < 0.05, **P <* *0.01 and ***P <* *0.001.

## Results

### Cytotoxicity of the DPDS derivatives in human cancer cell lines

**Figure 1:**
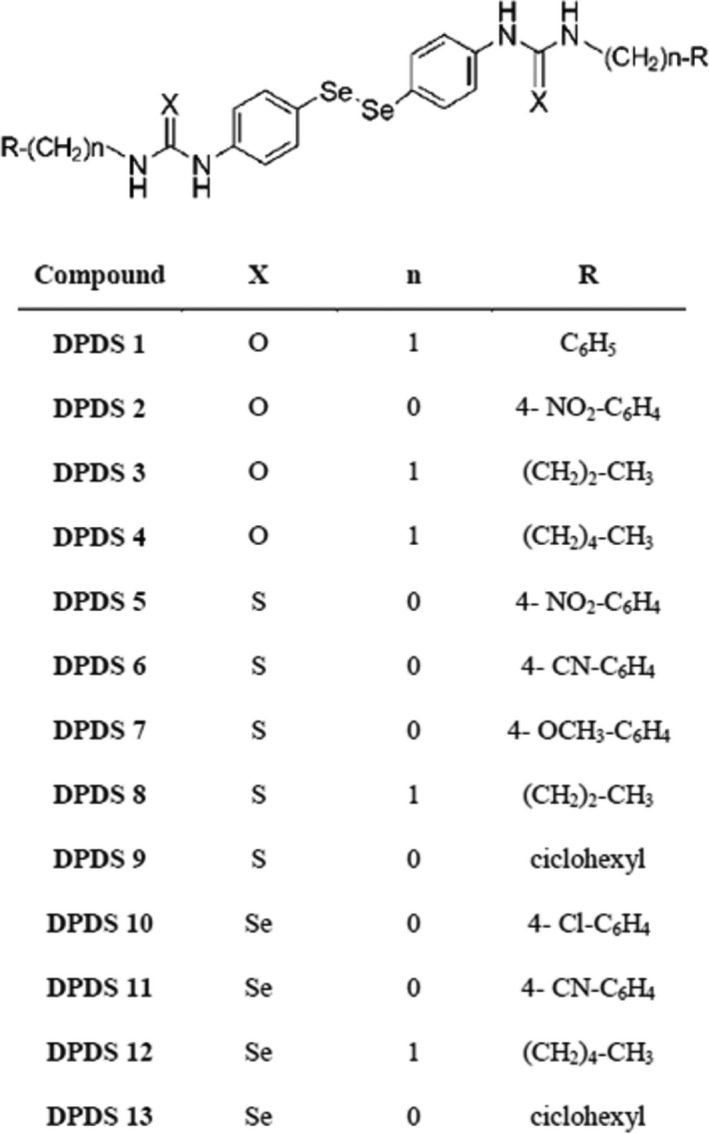
Structure of the DPDS used in this work.

The growth inhibitory activities of the thirteen DPDS‐related compounds, whose structures are shown in Figure 1, were determined in HTB‐54, HT‐29, MCF‐7, PC‐3, CCRF‐CEM and K‐562 cells. Assays were performed using the MTT method. The cytotoxic effect of each substance was tested at five different concentrations ranging from 0.01 to 100 μM. GI_50_, and LC_50_ values were calculated from dose‐response curves. Doxorubicin, camptothecin, etoposide and cisplatin were used as positive controls. As shown in Table 1, most of the tested compounds displayed important *in vitro* anticancer activity. PC‐3 and leucemia cell lines were generally more sensitive to the cytotoxic effect of these derivatives compared with colon and lung cancer cell lines. Furthermore, most of the tested compounds displayed similar or increased *in vitro* anticancer activity compared with the standard chemotherapeutic agents cisplatin and etoposide. In fact, each compound yielded reduced GI_50_ values compared with etoposide in HT‐29 and MCF‐7 cells. DPDSs **1**–**8**,** 11** and **12** also produced similar results in K‐562 cells. In addition, DPDSs **2**,** 5** and **6** in K‐562 cells; DPDSs **1–7** and **47** in PC‐3 cells; DPDSs **1**,** 3**,** 4** and **7** in HT‐29 cells; and DPDSs **2** and **5**–**7** in HTB‐54 cells exhibited reduced GI_50_ values compared with cisplatin.

**Table 1:** GI_50_ values (μM) for DPDS 1‐13

	K‐562	CCRF‐CEM	HT‐29	PC‐3	MCF‐7	HTB‐54	BEAS	184B5
1	8.73	9.81	5.71	1.64	8.78	20.34	0.05	1.51
2	1.91	0.30	8.71	1.97	5.89	3.10	0.62	0.07
3	7.11	6.36	0.95	0.04	10.94	21.40	0.04	0.06
4	8.75	13.50	6.83	1.12	8.36	28.76	0.002	0.18
5	1.00	2.12	22.68	4.33	6.66	6.53	2.83	2.69
6	3.18	2.38	9.42	4.57	4.61	3.27	15.79	2.93
7	6.98	6.68	6.36	4.30	4.52	5.41	4.03	5.08
8	9.30	9.27	14.31	10.23	10.47	29.57	6.77	5.99
9	20.3	19.42	24.54	24.42	16.06	31.06	17.29	3.39
10	13.7	2.71	10.33	7.01	5.16	24.06	6.38	5.39
11	5.57	2.04	19.73	6.13	8.43	26.62	9.24	5.24
12	8.55	1.69	15.75	3.87	7.24	16.04	22.54	5.14
13	12.9	2.67	14.91	9.33	12.56	26.44	10.11	5.84
Doxorubicin	0.10	<10^−2^	0.20	0.25	0.02	n.d	0.10	n.d
Camptothecin	0.06	n.d	0.05	0.06	0.01	n.d	0.06	n.d
Etoposide	12.5	1.58	31.62	0.63	19.95	n.d	12.5	n.d
Cisplatin	5.01	1.72	7.95	5.01	3.16	9.64	5.01	n.d

n.d.: not determined.

To determine the selectivity of these compounds for tumour cells, cytotoxicity was also assessed in the non‐malignant cell lines 184B5 and BEAS‐2B (Table 1). The selectivity index (SI) for breast and lung cells was calculated from LC_50_ values according to the formulas SI = LC_50_ (184B5)/LC_50_ (MCF‐7) and LC_50_ (BEAS‐2B)/LC_50_ (HTB‐54). Interestingly, although most of the analysed compounds exhibited low selectivity for malignant cells, DPDS **6** exhibited a high selectivity for tumour cells in lung cultures (LC_50_ BEAS‐2B = 78.77 μM; LC_50_ HTB‐54 = 10.31 μM; SI = 7.63).

### DPDS derivatives induce cell cycle arrest

**Figure 2:**
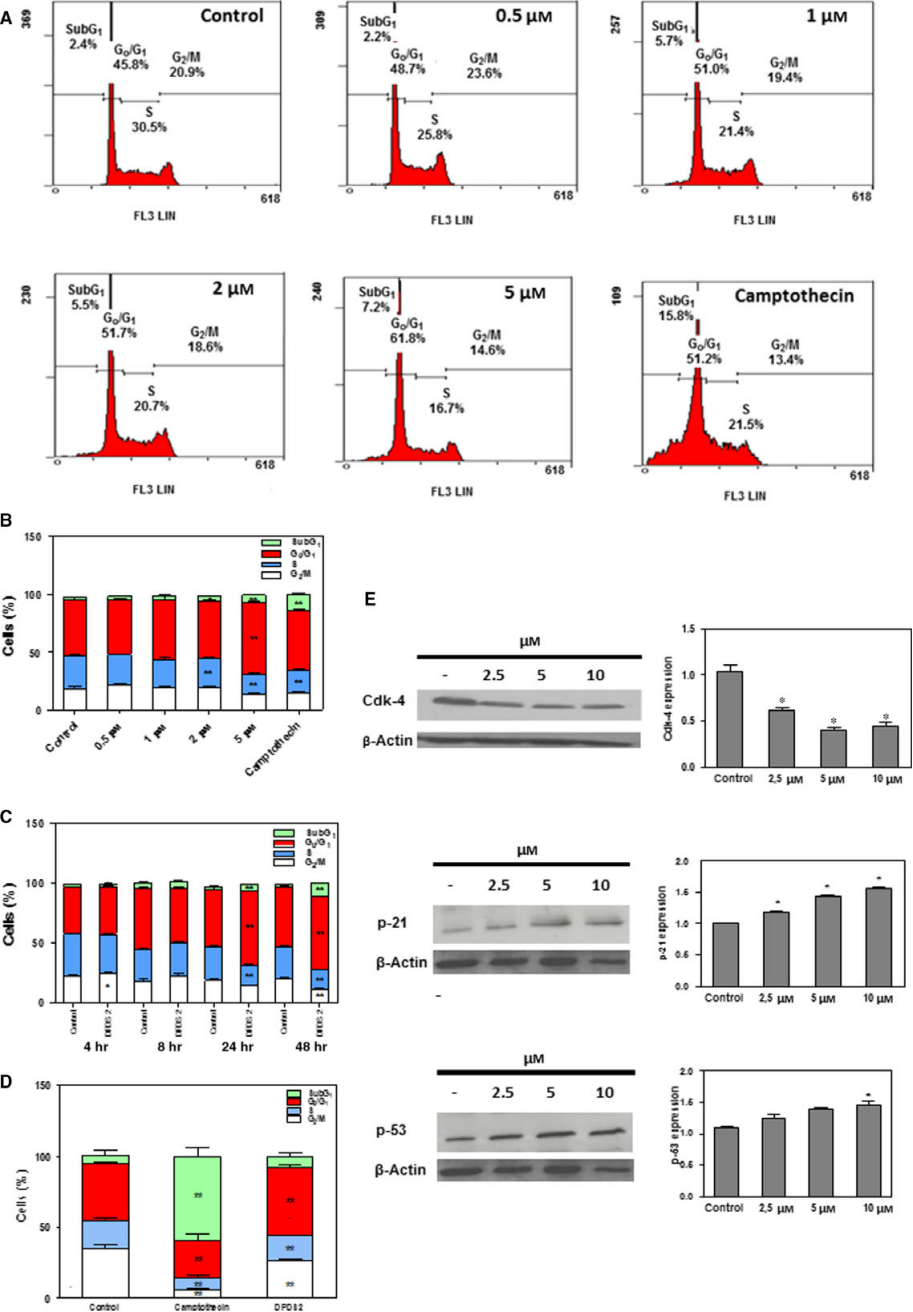
DPDS **2** induces G_0_/G_1_ cell cycle arrest in CCRF‐CEM cells. CCRF‐CEM cells were exposed to 0.5, 1, 2 and 5 μM DPDS **2** or vehicle for 24 hrs. After incubation, cells were processed for propidium iodide staining, and their cellular DNA content was determined by flow cytometry. Positive controls contained camptothecin at the same concentration as DPDS **2**. (**A**) Flow cytometry analysis of a representative experiment. (**B**) The results are expressed as percentages of total cell counts. (**C**) Time course analysis of cell cycle phase distribution after incubation of the cells with 5 μM of DPDS **2**. (**D**) Cell cycle phase distribution of MOLT‐4 cell cultures after treatment with 20 μM DPDS **2** for 24 hrs. (**E**) Western blot analysis of CDK4, p21^CIP1^ and p53 in CCRF‐CEM cells treated with 2.5, 5 and 10 μM of compound DPDS **2** for 24 hrs. The results are the mean ± S.E.M. of at least three independent experiments. *P < 0.05 and **P < 0.01 compared with control cells.

Given that cell cycle regulation is involved in the therapeutic effects of several Se‐containing compounds 29, 30 we next analysed whether the DPDS derivatives affected cell cycle progression. In these studies, DPDS **2**, the compound that yielded the lowest mean GI_50_ value across all cell lines (ranging from 0.30 to 8.71 μM) in the panel, and DPDS **6**, the compound with the highest SI in lung cells, were chosen. Given that T‐ALL CCRF‐CEM cells were the most sensitive cells towards the majority of the tested compounds, DPDS **2** action on cell cycle was assessed by determining the cell cycle phase distribution of CCRF‐CEM cells after treatment with different concentrations of DPDS **2** for 24 hrs. Determinations were performed by measuring the DNA content of the cells by propidium iodide staining and flow cytometry. Camptothecin was used as a positive control. Results are presented in Figure 2A and B. According to the cytotoxic effects exerted by DPDS **2**, a significant increase in the hypodiploid SubG_1_ cell population was detected after treatment with 2 μM or greater DPDS **2**. Consistent with cell cycle arrest at the G_0_/G_1_ phase, a significant increase in the G_0_/G_1_ cell population accompanied by a significant reduction in the percentage of cells in both G_2_/M and S phases was observed with 5 μM DPDS **2**. Time course analysis of cell cycle distribution upon treatment with 5 μM DPDS **2** confirmed G_0_/G_1_ cell cycle arrest at 24 and 48 hrs (Fig. 2C). The ability of DPDS **2** to induce cell cycle arrest at the G_0_/G_1_ phase was corroborated in T‐ALL MOLT‐4 cells (Fig. 2D). According to the Sanger Institute Catalogue of Somatic Mutations in Cancer (COSMIC; http://www.sanger.ac.uk/cosmic), CCRF‐CEM cells carry a homozygous deletion of the *CDKN2A* locus (c.1_471del471), which affects p16^INK4^, a CDK4‐specific inhibitor. In contrast, wild‐type p53 down‐regulates CDK4 translation 31. Thus, to further examine the mechanism by which DPDS **2**‐induced G_0_/G_1_ cell cycle arrest, the expressions of CDK4, p14^ARF^, p53 and p21^CIP1^ proteins in CCRF‐CEM cells treated with 2.5, 5 or 10 μM DPDS **2** for 24 hrs were determined by Western blot. Consistent with DPDS **2**‐mediated cell cycle arrest at G_0_/G_1_, CDK4 expression was markedly decreased after the treatments (Fig. 2E). In addition, treated CCRF‐CEM cells exhibited increased levels of p21^CIP1^ (Fig. 2E) and p53 (Fig. 2E) tumour suppressor proteins, whereas p14^ARF^ could not be detected in these *CDNK2A*‐defective cells (not shown).

**Figure 3:**
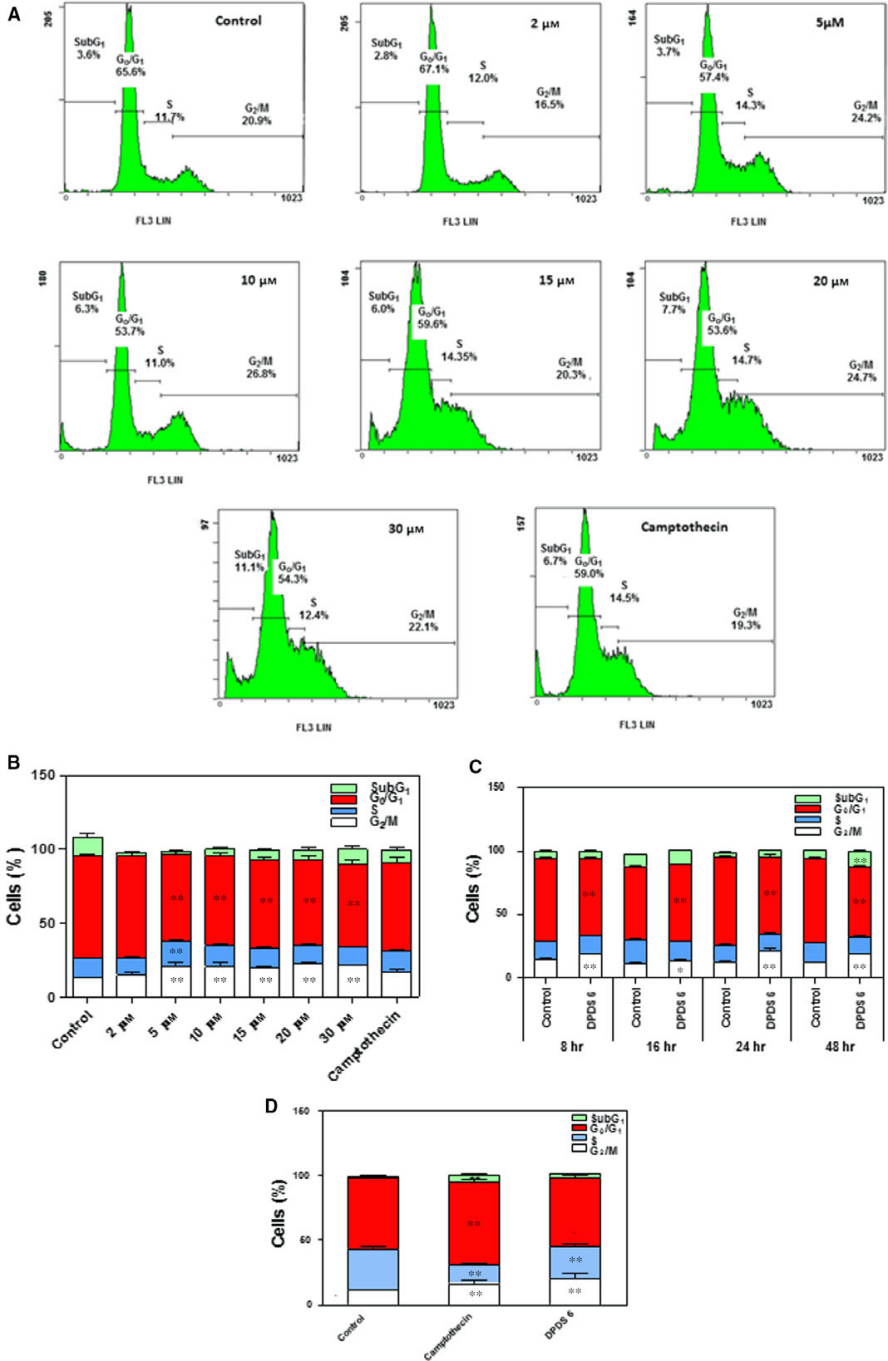
DPDS **6** induces G_2_/M cell cycle arrest in HTB‐54 cells. HTB‐54 cells were exposed to 2, 5, 10, 15, 20 and 30 μM DPDS **6** or vehicle for 24 hrs. After incubation, cells were processed for propidium iodide staining, and their cellular DNA content was determined by flow cytometry. Positive controls contained camptothecin at the same concentration as DPDS **6**. (**A**) Flow cytometry analysis of a representative experiment. (**B**) The results expressed as percentages of total cell counts. (**C**) Time course analysis of cell cycle phase distribution after incubation of the cells with 10 μM DPDS **6**. (**D**) Cell cycle phase distribution of A549 cell cultures after treatment with 30 μM DPDS **6** for 24 hrs. The results are the mean ± S.E.M. of three independent experiments performed in duplicate. *P < 0.05, **P < 0.01 and ***P < 0.001 compared with control cells.

To assess the effect of DPDS **6** on cell cycle progression in lung cells, HTB‐54 cell cultures were exposed to 2, 5, 10, 15, 20 or 30 μM DPDS **6** or vehicle (control cells) for 24 hrs, and the cell cycle phase distribution of the cultures was determined as described above. A significant decrease in the G_0_/G_1_ cell population and an increase in the percentage of G_2_/M cells were detected after treatment with 5 μM or greater DPDS **6** (Fig. 3A and B). Thus, these results suggest that DPDS **6** induces cell cycle arrest in the late G_2_/M phase. Time course analysis of cell cycle distribution upon treatment with 10 μM of DPDS **6** confirmed DPDS **6**‐mediated cell cycle arrest as soon as 8 hrs (Fig. 3C). The ability of DPDS **6** to induce cell cycle arrest at G_2_/M in lung cancer cells was confirmed in A549 cell cultures (Fig. 3D).

### DPDS 2 triggers caspase‐dependent apoptosis in CCRF‐CEM cells

**Figure 4:**
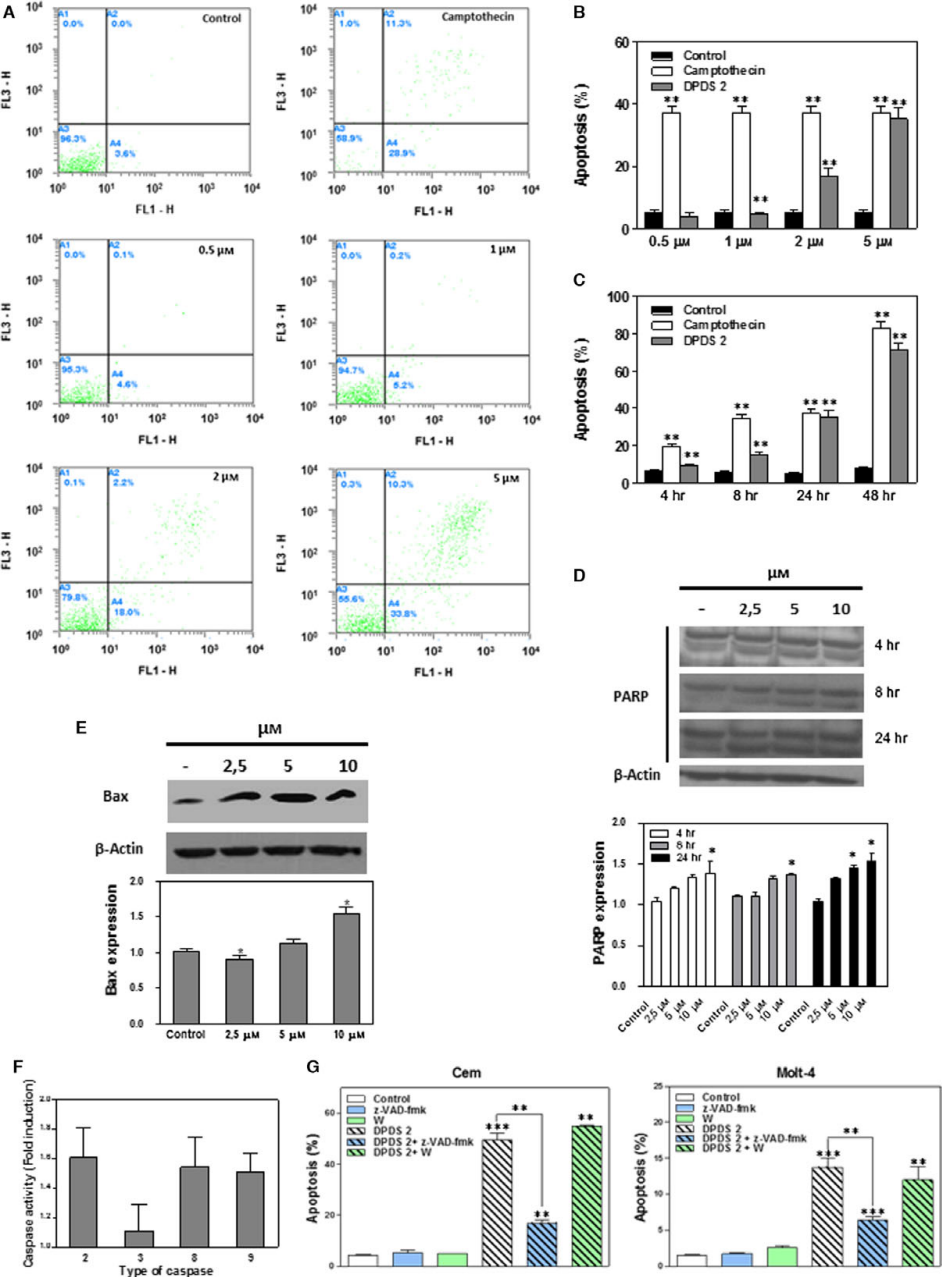
DPDS **2** induces caspase‐dependent cell death in CCRF‐CEM cells. CCRF‐CEM cells were exposed to 0.5, 1, 2 and 5 μM DPDS **2** or vehicle for 24 hrs. After incubation, the rate of apoptotic cell death was determined by Annexin V‐FITC staining. Positive controls contained camptothecin at the same concentration as DPDS **2**. (**A**) Flow cytometry analysis of a representative experiment. (**B**) The results expressed as percentages of apoptotic cells in the cultures. (**C**) Time course analysis of DPDS **2**‐induced cell death; CCRF‐CEM cells were treated with 5 μM DPDS **2** for different periods. (**D**) PARP and (**E**) BAX protein levels in CCRF‐CEM cells treated with 2.5, 5 and 10 μM DPDS **2** or vehicle for the indicated time periods were determined by Western blot. (**F**) CCRF‐CEM cells were incubated with either 5 μM DPDS **2** or vehicle (control cells) for 24 hrs. After incubation, caspase‐2, ‐3, ‐8 and ‐9 activities were determined. Data are presented as the ratio between caspase activities in DPDS **2**‐treated and untreated cells. (**G**) For cell death inhibition in CCRF‐CEM and MOLT‐4 cells, z‐VAD‐fmk (50 μM) and wortmannin (10 μM) were added to the culture medium 1 hr before DPDS **2** treatment. The results are the mean ± S.E.M. of three independent experiments. *P < 0.05 and **P < 0.01 compared with control cells.

Apoptosis forms the core of the anticancer effects displayed by Se compounds 32. Therefore, we sought to determine the role of apoptosis in the cytotoxicity exerted by both DPDS **2** and DPDS **6** in CCRF‐CEM and HTB‐54 cells, respectively. Therefore, CCRF‐CEM cells were incubated in the presence or absence (control) of 0.5, 1, 2 or 5 μM DPDS **2** for 24 hrs, and the apoptotic status of the cells was determined by measuring phosphatidylserine exposure on the cell membranes using the *Annexin V‐FITC Kit*. Camptothecin was used as a positive control. The results are shown in Figure 4A and B. DPDS **2** triggered apoptosis in CCRF‐CEM cells in a concentration‐dependent manner. Moreover, when cells were treated with 5 μM DPDS **2** for different time periods, enhancement of cell death was detected as early as 4 hrs, and the number of dead cells increased with time (Fig. 4C). These results indicated that DPDS **2**‐mediated apoptosis is also time dependent. Consistent with these results, blot analysis demonstrated the accumulation of cleaved PARP in CCRF‐CEM cells after 4, 8 and 24 hrs of treatment with DPDS **2** (Fig. 4D). This accumulation was observed at every DPDS **2** concentration tested. Interestingly, BAX was also increased in CCRF‐CEM cells after 24 hrs of treatment with 10 μM DPDS **2** (Fig. 4E). Similarly, caspase‐2, ‐3, ‐8 and ‐9 (Fig. 4F) activity levels increased in CCRF‐CEM cells after treatment. In addition, pre‐treatment of the cells with the pancaspase inhibitor z‐VAD‐fmk protected both CCRF‐CEM and MOLT‐4 cells from DPDS **2**‐induced apoptosis (Fig. 4G). However, pre‐treatment with the PI3K inhibitor wortmannin did not prevent DPDS **2**‐induced cell death in these cells (Fig. 4G). Taken together, these results suggest a prominent role for caspases in DPDS **2**‐mediated apoptosis in T‐ALL cells.

**Figure 5:**
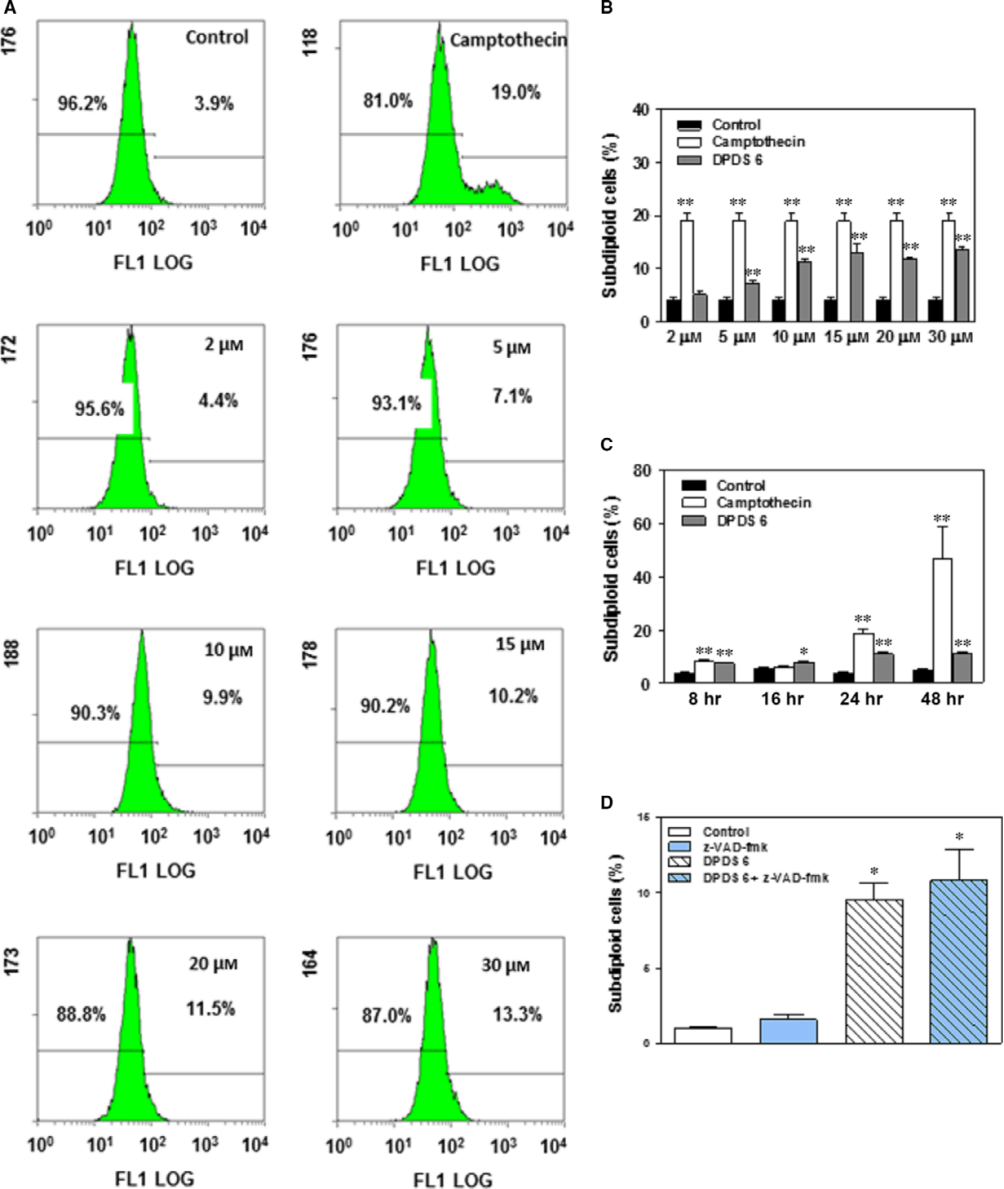
DPDS **6**‐induced cell death in HTB‐54 cell cultures is independent of caspase activation. HTB‐54 cells were exposed to 2, 5, 10, 15, 20 and 30 μM DPDS **6** or vehicle for 24 hrs. After incubation, cell death was determined by TUNEL. Positive controls contained camptothecin at the same concentration as DPDS **6**. (**A**) Flow cytometry analysis of a representative experiment. (**B**) The results expressed as percentages of dead cells in the cultures. Subdiploid cells were considered apoptotic. (**C**) Time course analysis of HTB‐54 cell death after treatment with 10 μM DPDS **6**. (**D**) HTB‐54 cells were pre‐treated with 50 μM z‐VAD‐fmk for 1 hr and incubated with either 10 μM DPDS **6** or vehicle for 24 hrs before cell death determination. The results are the mean ± S.E.M. of three independent experiments. *P < 0.05 and **P < 0.01 compared with control cells.

To test the ability of DPDS **6** to induce apoptosis, HTB‐54 cells were incubated in the presence or absence (control) of 2, 5, 10, 15, 20 or 30 μM DPDS **6** for 24 hrs, and the apoptotic status of the cells was determined using the Apo‐Direct Kit, which is based on the TUNEL technique. As shown in Figure 5A and B, a slight increase in DNA degradation was detected after treatment with 5 μM or higher DPDS **6**. In addition, this increase was concentration dependent but not time dependent (Fig. 5C). Furthermore, the pro‐apoptotic effects of DPDS **6** were markedly reduced compared with those exerted by camptothecin, even after incubation of HTB‐54 cells with high doses of DPDS **6** (10–30 μM) for 24 hrs (Fig. 5B) or for longer periods of time (Fig. 5C). Finally, induction of cell death by DPDS **6** in HTB‐54 cells could not be prevented by pre‐treatment with the pancaspase inhibitor z‐VAD‐fmk (Fig. 5D). Therefore, the data suggest that caspase‐dependent apoptosis is not the main cell death pathway involved the cytotoxic effect triggered by DPDS **6** in HTB‐54 cells.

### DPDS 6 induces an autophagic cell death process in HTB‐54 cells

**Figure 6:**
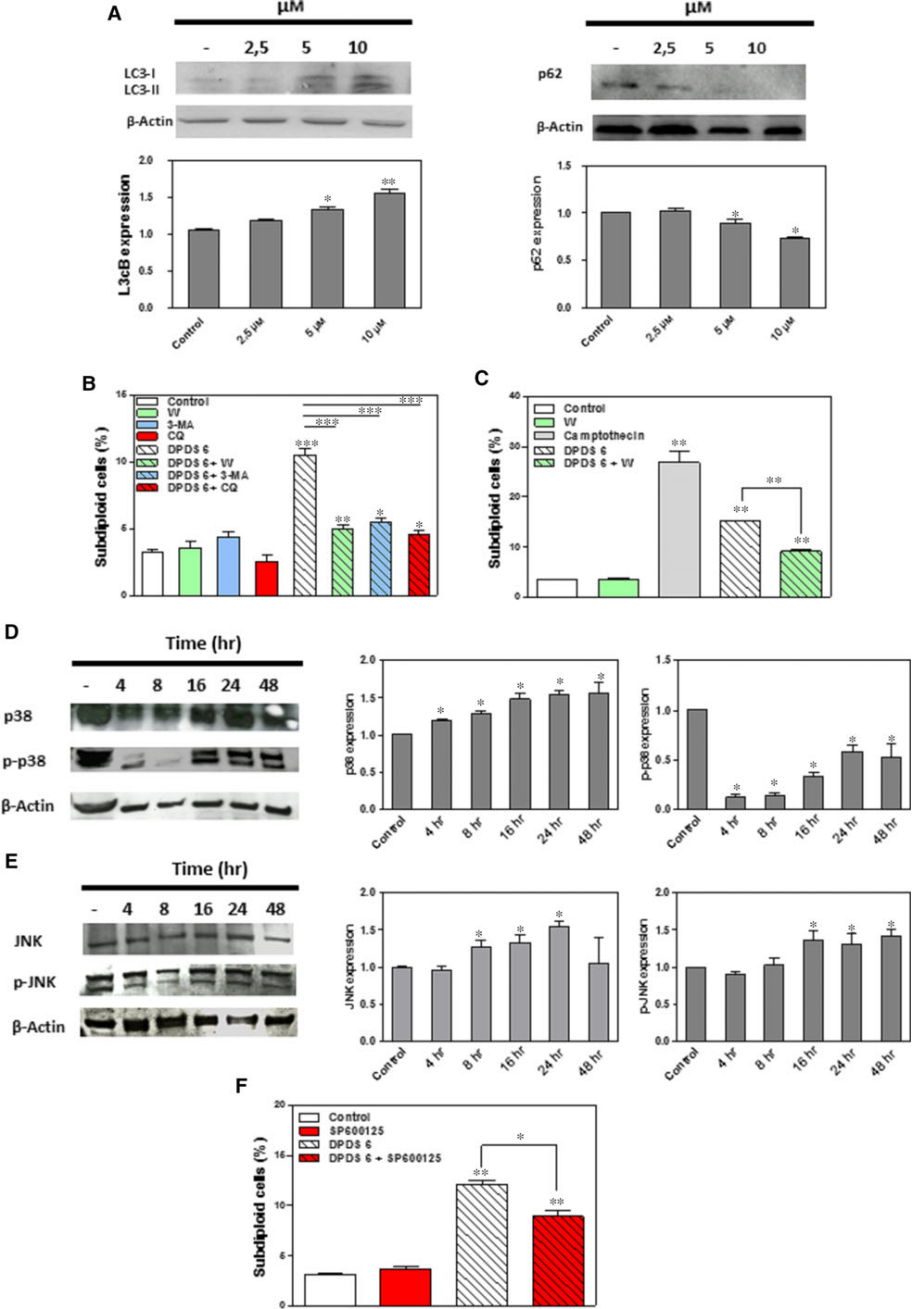
DPDS **6** induces autophagy in HTB‐54 cells. (**A**) Western blot analysis of SQTMS1/p62 and LC3 in HTB‐54 cells treated with 2.5, 5 and 10 μM DPDS **6** or vehicle for 24 hrs. (**B**) HTB‐54 cells pre‐treated with 5 mM 3‐MA, 10 μM wortmannin or 10 μM CQ for 1 hr were incubated with either 10 μM DPDS **6** or vehicle for 24 hrs. Then, cell death was determined by TUNEL. (**C**) Cell death determination in A549 cell cultures after exposure to 30 μM DPDS **6** either in the presence or absence of 10 μM wortmannin. (**D**) Time course analysis of p38 and p‐p38 MAPK expression in HTB‐54 cells exposed to 10 μM DPDS **6**. (**E**) Time course analysis of JNK and p‐JNK expression in HTB‐54 cells exposed to 10 μM of DPDS **6**. (**F**) HTB‐54 cells pre‐treated with 10 μM SP600125 for 1 hr were incubated with 10 μM DPDS **6** or vehicle for 24 hrs. Then, cell death was determined by TUNEL. The results are the mean ± S.E.M. of three independent experiments. *P < 0.05 and **P < 0.01 compared with control cells.

Autophagic cell death induction by some anticancer agents has been proposed as a new cancer treatment modality 33, 34. Hence, to further analyse the molecular mechanism by which DPDS **6** reduced HTB‐54 cell viability, we explored the significance of autophagy in this process. As shown in Figure 6A, a reduction in SQTMS1/p62 and increased LC3‐II levels 35 were detected in HTB‐54 cells after exposure to 5 μM or greater DPDS **6** for 24 hrs. In addition, pre‐incubation of the cells with the autophagy inhibitors 3‐MA, wortmannin and CQ 36 markedly reduced DPDS **6**‐induced cell death (Fig. 6B). Wortmannin also protected A549 cells from DPDS **6**‐induced cell death (Fig. 6C). In contrast, wortmannin could not prevent induction of CCRF‐CEM cell death by DPDS **2** (Fig. 4G).

Given that JNK and p38 MAPK signallings are implicated in the control of the balance of autophagy and apoptosis in response to chemotherapeutic agents 37, we evaluated whether DPDS **6**‐mediated autophagy involves JNK or p38 MAPK activation in HTB‐54 cells. JNK, p‐JNK, p38 MAPK and p‐p38 MAPK expression levels were determined by Western blot in HTB‐54 cells after treatment with 10 μM DPDS **6** for different time periods. As shown in Figure 6D and E, increased levels of p38 and JNK were detected after 4 and 8 hrs, respectively. However, although increased levels of p‐JNK were observed after 16 hrs of exposure to DPDS **6**, a reduction in p‐p38 MAPK was obvious at 4 hrs and remained low throughout the experiment. In addition, pre‐incubation of the cells with the broad‐spectrum JNK inhibitor SP600125‐protected HTB‐54 cells from DPDS **6**‐induced autophagy (Fig. 6F). Taken together, these results suggest that DPDS **6** induces autophagic cell death in HTB‐54 cells, which is characterized by JNK up‐regulation and p38 MAPK signalling inhibition.

## Discussion

Converging lines of evidence demonstrate that Se compounds have potential chemotherapeutic effects in a range of cancers. These derivatives can be dosed in a variety of chemical forms, each of which may display different anticancer mechanisms. In the present study, the anticancer effects of novel DPDS derivatives were evaluated. In general, most of the tested compounds exhibited potent cytotoxic effects against a series of human cancer cell lines. Among the tested compounds, derivatives **2** and **6** exhibited the most potent anticancer activity. Hence, we also explored the molecular mechanism involved in their tumour‐mediated cytotoxicity. We found that DPDS **2**‐induced apoptosis in leucemia CCRF‐CEM cells, whereas autophagy was the main cell death pathway triggered by DPDS **6** in HTB‐54 lung cancer cells.

Currently, major efforts are focused on the design of strategies to induce cell growth arrest and apoptosis selectively in tumour cells. Numerous signals, including DNA damage and oxidative stress activate p53 38. Regardless of the signal, p53 activation might lead to either cell cycle arrest *via* p21^CIP1^ transcription or to apoptosis by inducing transcription of proteins, such as BAX, NOXA and PUMA 39, and physically interacting with BCL‐2 family members to promote mitochondrial outer membrane permeabilization 40. Of note, we observed increased levels of the p53 tumour suppressor protein in T‐ALL CCRF‐CEM cells treated with DPDS **2**. Although not frequently noted at diagnosis, p53 mutations are associated with poor clinical outcome in T‐ALL 41. Constitutive activation of *NOTCH1* signalling and loss of the *CDKN2A* locus, which encompasses the cell cycle regulators p16^INK4^ and p14^ARF^, is the most frequently reported alterations implicated in the development of T‐ALL 42. In fact, inactivation of the *CDKN2A* gene by deletion, mutation or promoter hypermethylation is noted in up to 90% of T‐ALL cases 41. Epigenetic inactivation of p21^CIP1^ has also been described in T‐ALL samples 43. As a result, the p53 pathway has been proposed as a possible target for therapy in T‐ALL 41. Hence, our finding of increased levels of the p53 protein in the *CDNK2A*‐defective CCRF‐CEM cell line after treatment with DPDS **2** is of great relevance. Furthermore, consistent with p53 up‐regulation, we also observed G_0_/G_1_ cell cycle arrest at the G_0_/G_1_ phase and increased expression of the p21^CIP1^ cell cycle regulator in CCRF‐CEM cells after treatment with DPDS **2**. Given that we also observed increased expression of BAX protein, our results indicate that DPDS **2** induces both cell cycle arrest and activation of the intrinsic apoptotic pathway. Further supporting the notion that DPDS **2** as an activator of the intrinsic apoptotic pathway; enhanced caspase‐9 activity and increased PARP cleavage were detected in CCRF‐CEM cells after treatment with this compound. Additionally, caspase‐8, an essential protease involved in the activation of the extrinsic apoptotic pathway, was also increased in DPDS **2**‐treated CCRF‐CEM cells. This result might reflect different aspects of the apoptotic process displayed by DPDS **2**. Indeed, several studies demonstrate that extrinsic and intrinsic pathways of caspase activation are extensively interconnected, and caspase‐8 is activated by caspase‐9 in several experimental systems 44.

A common feature of organic Se compounds is that small changes in their structure can lead to dramatic changes in their biological effects. Thus, it is not striking that compounds **2** and **6**, which are both DPDS analogues, displayed different mechanisms of action. In fact, when tested in non‐malignant BEAS‐2B and HTB‐54 non‐small lung cancer cells, DPDS **6** exhibited a strong toxicity and selectivity for tumour cells. Given that HTB‐54 is a p53 null cell line (http://p53.free.fr), this result implies that DPDS **6** induces a cell death programme that differs from p53‐dependent apoptosis. Autophagy is a conserved lysosomal degradation pathway that is involved in both non‐apoptotic tumour cell death and paradoxically tumour progression. In addition to apoptosis, induction of autophagy has been described among the cellular and molecular mechanisms involved in the anticancer activity of several seleno‐compounds. For instance, chronic exposure to supranutritional doses of sodium selenite increase autophagy in normal colonic fibroblasts 45, whereas autophagy and apoptosis are both engaged in selenite‐induced cell death in Jurkat 6 and NB4 7 cells. Similar to other pro‐autophagic anticancer agents, DPDS **6** inhibited the cell cycle, induced a caspase‐independent cell death programme, reduced SQTMS1/p62 levels and an increased LC3‐II levels in HTB‐54 cells. Moreover, the cytotoxic effects of DPDS **6** in HTB‐54 cells were abolished by the presence of PI3K inhibitors (3‐MA or wortmannin) or a lysosomotropic agent (CQ) 36, thus suggesting a pivotal role for autophagy in the anticancer effects exerted by DPDS **6**. Although crosstalk between autophagy and apoptosis has not been completely deciphered, several lines of evidence suggest that both pathways are interconnected and even simultaneously activated in neoplastic cells 15. In fact, concurrent apoptosis and autophagy have been detected in selenite‐treated colon cancer cells. Interestingly, both basal and selenite‐induced autophagy levels varied depending on the p53 status of the cells. These levels were increased when this protein was deleted 23. Thus, our finding that DPDS **6** induces autophagy in p53 null HTB‐54 lung cancer cells is not surprising.

Over recent years, several reports have implicated JNK and p38 MAPK pathways in regulating the balance between apoptosis and autophagy in response to chemotherapeutic agents 17, 37, 45, 46, 47. p38 MAPK has a dual role as an activator and inhibitor of autophagy 37. When activated by the GADD45B‐MAP3K4 signalling complex, p38 MAPK is directed to autophagosomes where it phosphorylates Atg5, impairing autophagy 48. A direct competition between Atg9, a transmembrane protein required for autophagy, and p38 MAPK for binding to p38 IP was also reported to inhibit autophagy 46. In contrast, JNK1 activation under starvation conditions promotes Bcl‐2/Bcl‐xL phosphorylation, which results in the release of Beclin 1 from the Beclin 1‐Bcl‐2/Bcl‐xL complex. At first, the liberation of Beclin 1 up‐regulates LC3 and stimulates autophagy. After prolonged phosphorylation, autophagy can no longer protect cells, and apoptosis is induced 17, 49. Interestingly, sodium selenite anticancer effects are associated with JNK activation in colon cancer cell lines 8 and p38 MAPK modulation in Jurkat and NB4 cells 6, 7. Modulation of p38 MAPK activity using a combination of aspirin and ABT‐737 has also been demonstrated in A549 and H1299 lung cancer cells 47. Consistent with these observations, we demonstrated a reduction in p‐p38 MAPK and an increase in p‐JNK levels in HTB‐54 cells after treatment with DPDS **6**. Furthermore, SP600125, a compound that inhibits JNK phosphorylation in Jurkat T cells 50 and induces p38 MAPK phosphorylation in mouse pancreatic beta cells MIN6 51, prevented induction of autophagy by DPDS‐**6**. Our results thus suggest that DPDS **6** triggers autophagic cell death in p53‐deficient lung cancer cells through the modulation of p38 MAPK and JNK signalling pathways. The potent anticancer effects and the ability of DPDS **6** to spare non‐malignant cells suggest that the balance between apoptosis and autophagy is optimal under these experimental circumstances. Regarding the structure‐activity relationship of this compound, a recent study proposes the *N,N′‐*diarylurea motif as a potent p38 MAPK inhibitor 52. However, further experiments, including the design and synthesis of structurally modified DPDS **6** derivatives are required to dissect the role of Se and *N,N′‐*diarylurea motifs in the regulation of JNK and p38 MAPK pathways by this compound.

In summary, the present study demonstrates the anticancer activity of a variety of novel DPDS derivatives. Overall, these compounds displayed potent *in vitro* cytotoxic effects against tumour cells. DPDS **2** and **6**, which are the most potent derivatives, exhibited strong anticancer activities through the induction of apoptosis and autophagy, respectively. When using normal and lung cancer cell lines, DPDS **6** also displayed a strong selectivity for p53‐deficient cell lines, where it modulates JNK and p38 MAPK activities. Whether DPDS **6** administration could result in a better outcome in the treatment of impaired p53‐bearing lung cancer cells merits further research.

## Conflicts of interest

The authors confirm that there are no conflict of interests.
